# Supplementary material for: Impact of IRS: Four-years of entomological surveillance of the Indian Visceral Leishmaniases elimination programme
Source: PLoS Negl Trop Dis. 2021 Aug 9;15(8):e0009101. doi: 10.1371/journal.pntd.0009101 (PMC8376195; doi:10.1371/journal.pntd.0009101)
Supplement: S1 Table — (DOCX) [file pntd.0009101.s001.docx]

| **District** | **Block** | **Village** | **IRS status** | **Collection dates** | **Comments** |
| --- | --- | --- | --- | --- | --- |
| Darjeeling | Phansidewa | Madhavita | IRS | 11/17 to 12/19 |  |
|  |  | Moonee div. | IRS | 11/17 to 12/19 |  |
|  |  | Motidhar T.E | IRS | 11/17 to 12/19 |  |
|  |  | Taipoo T.E | IRS | 11/17 to 12/19 |  |
|  |  | Kalamgachh | Non-irs | 11/17 to 12/19 |  |
|  |  | Piapukur | Non-irs | 11/17 to 12/19 |  |
| East Champaran | Turkauliya | Jaisinghpur north | IRS | 10/17 to 12/19 |  |
|  |  | madhopur | IRS | 10/17 to 12/19 |  |
|  |  | Madhumalat | IRS | 10/17 to 12/19 |  |
|  |  | Mathurapur | IRS | 10/17 to 12/19 |  |
|  |  | Bijulpur | Non-irs | 10/17 to 12/19 |  |
|  |  | Chainpur | Non-irs | 10/17 to 12/19 |  |
| Godda | Poraiya Haat | Bhatonda | IRS | 01/17 to 12/19 |  |
|  |  | Gumma | IRS | 01/17 to 12/19 |  |
|  |  | Kathon | IRS | 01/17 to 12/19 |  |
|  |  | Sakri | IRS | 01/17 to 12/19 |  |
|  |  | Baxara | Non-irs | 01/17 to 12/19 |  |
|  |  | Birniya | Non-irs | 01/17 to 12/19 |  |
| Gopalganj | Barauli | Barauli | IRS | 10/16 to 12/19 |  |
|  |  | Kalyanpurmathiya | IRS | 10/16 to 12/19 |  |
|  |  | Rupanchap | IRS | 10/16 to 12/19 |  |
|  |  | Sadaua | IRS | 10/16 to 12/19 |  |
|  |  | Sarar | Non-irs | 10/16 to 06/19 | sprayed in response to spike in cases |
|  |  |  | Non-irs | 06/19 to12/19 |  |
|  |  | Jokaha | IRS | 10/16 to 04/19  11/19 to12/19 | sprayed in response to spike in cases |
|  |  |  | IRS | 04/19 to 11/19 |  |
| Katihar | Barari | Balua | IRS | 10/16 to 12/19 |  |
|  |  | Kajra | IRS | 10/16 to 12/19 |  |
|  |  | Kawar-kothi | IRS | 10/16 to 12/19 |  |
|  |  | Siwana | IRS | 10/16 to 12/19 |  |
|  |  | Ghuski | Non-irs | 10/16 to 12/19 |  |
|  |  | Milik tola | Non-irs | 10/16 to 12/19 |  |
| Muzaffarpur | Minapur | Alineora | IRS | 04/16 to 03/18 |  |
|  |  |  | Non-IRS | 04/18 to 12/19 |  |
|  |  | Bajarmuriya | IRS | 04/18 to 12/19 | new village added |
|  |  | Chandparna | IRS | 04/16 to 12/19 |  |
|  |  | Maksoodpur | IRS | 04/16 to 03/18 |  |
|  |  |  | Non-IRS | 12/18 to 12/19 |  |
|  |  | Minapur | IRS | 04/16 to 12/19 |  |
|  |  | Bahwal | Non-IRS | 04/16 to 12/19 |  |
|  |  | Chakimaad | Non-IRS | 04/16 to 12/19 |  |
| Purnia | Dhamdaha | Bishanpur | IRS | 10/16 to 12/19 |  |
|  |  | Dhamdaha uttar | IRS | 04/16 to 12/19 |  |
|  |  | Dharharjamuniya | IRS | 04/16 to 12/19 |  |
|  |  | Kajra | IRS | 04/16 to 12/19 |  |
|  |  | Kukron | Non-IRS | 10/16 to 11/17 |  |
|  |  |  | IRS | 12/17 to 12/19 |  |
|  |  | Parasmani | Non-IRS | 10/16 to 12/16 |  |
|  |  |  | IRS | 01/17 to 12/19 |  |
| Samastipur | Warishnagar | Balahi | IRS | 04/16 to 12/19 |  |
|  |  | Dhanhar | IRS | 04/16 to 12/19 |  |
|  |  | Kusaiya | IRS | 04/16 to 12/19 |  |
|  |  | Rahua east | IRS | 04/16 to 03/17 |  |
|  |  |  | Non-IRS | 03/17 to 12/19 |  |
|  |  | Chandauli | Non-IRS | 04/16 to 12/19 |  |
|  |  | Kashor | Non-IRS | 04/16 to 12/19 |  |
