# Supplementary material for: Impact of IRS: Four-years of entomological surveillance of the Indian Visceral Leishmaniases elimination programme
Source: PLoS Negl Trop Dis. 2021 Aug 9;15(8):e0009101. doi: 10.1371/journal.pntd.0009101 (PMC8376195; doi:10.1371/journal.pntd.0009101)
Supplement: S2 Table — (DOCX) [file pntd.0009101.s002.docx]

| **Year** | **IRS Round** | **District** | **Rooms** | | | | | | | | |
| --- | --- | --- | --- | --- | --- | --- | --- | --- | --- | --- | --- |
|  |  |  | **Sprayed (Complete)** | | **Sprayed (Partial)** | | **Refused** | | **Locked** | | **Total** |
|  |  |  | **%** | **n** | **%** | **n** | **%** | **n** | **%** | **n** |  |
| **2017** | **1** | East Champaran | 95.97 | 15,335 | 0.00 | 0 | 2.62 | 419 | 1.41 | 225.00 | 15,979 |
|  |  | Godda | 83.86 | 6,589 | 2.02 | 159 | 14.11 | 1,109 | 0.00 | 0.00 | 7,857 |
|  |  | Gopalganj | 90.92 | 2,114 | 0.00 | 0 | 9.08 | 211 | 0.00 | 0.00 | 2,325 |
|  |  | Katihar | 98.36 | 9,548 | 0.00 | 0 | 1.04 | 101 | 0.60 | 58.00 | 9,707 |
|  |  | Muzaffarpur | 95.92 | 12,701 | 0.00 | 0 | 2.48 | 328 | 1.60 | 212.00 | 13,241 |
|  |  | Purnia | 95.70 | 10,619 | 0.00 | 0 | 2.53 | 281 | 1.77 | 196.00 | 11,096 |
|  |  | Samastipur | 93.61 | 10,826 | 0.00 | 0 | 4.23 | 489 | 2.16 | 250.00 | 11,565 |
|  | **2** | Darjeeling | 97.65 | 1,081 | 0.00 | 0 | 1.45 | 16 | 0.90 | 10.00 | 1,107 |
|  |  | East Champaran | 79.35 | 1,975 | 0.00 | 0 | 12.86 | 320 | 7.79 | 194.00 | 2,489 |
|  |  | Godda | 84.76 | 6,466 | 0.00 | 0 | 9.39 | 716 | 5.86 | 447.00 | 7,629 |
|  |  | Gopalganj | 92.41 | 6,059 | 0.00 | 0 | 7.59 | 498 | 0.00 | 0.00 | 6,557 |
|  |  | Muzaffarpur | 96.00 | 12,034 | 0.00 | 0 | 1.78 | 223 | 2.23 | 279.00 | 12,536 |
|  |  | Purnia | 96.44 | 14,931 | 0.00 | 0 | 2.40 | 372 | 1.16 | 179.00 | 15,482 |
|  |  | Samastipur | 93.11 | 13,061 | 0.00 | 0 | 3.64 | 511 | 3.25 | 456.00 | 14,028 |
| **2018** | **1** | Darjeeling | 99.63 | 4,838 | 0.00 | 0 | 0.08 | 4 | 0.29 | 14.00 | 4,856 |
|  |  | East Champaran | 96.94 | 13,549 | 0.00 | 0 | 2.15 | 301 | 0.90 | 126.00 | 13,976 |
|  |  | Godda | 80.49 | 5,404 | 0.00 | 0 | 9.47 | 636 | 10.04 | 674.00 | 6,714 |
|  |  | Gopalganj | 94.99 | 7,074 | 0.00 | 0 | 5.00 | 372 | 0.01 | 1.00 | 7,447 |
|  |  | Katihar | 98.67 | 5,413 | 0.00 | 0 | 0.86 | 47 | 0.47 | 26.00 | 5,486 |
|  |  | Muzaffarpur | 94.88 | 6,469 | 0.00 | 0 | 1.54 | 105 | 3.58 | 244.00 | 6,818 |
|  |  | Purnia | 96.05 | 14,576 | 0.00 | 0 | 2.43 | 369 | 1.52 | 230.00 | 15,175 |
|  |  | Samastipur | 93.90 | 12,445 | 0.00 | 0 | 3.45 | 457 | 2.65 | 351.00 | 13,253 |
| **2019** | **1** | Godda | 86.44 | 7,169 | 0.00 | 0 | 7.99 | 663 | 5.57 | 462.00 | 8,294 |
|  |  | Gopalganj | 94.35 | 5,089 | 0.00 | 0 | 3.13 | 169 | 2.52 | 136.00 | 5,394 |
|  |  | Purnia | 94.85 | 17,882 | 0.00 | 0 | 3.18 | 600 | 1.97 | 371.00 | 18,853 |
|  | **2** | East Champaran | 95.27 | 16,088 | 0.00 | 0 | 1.53 | 259 | 3.19 | 539.00 | 16,886 |
|  |  | Godda | 87.63 | 6,985 | 0.00 | 0 | 7.39 | 589 | 4.98 | 397.00 | 7,971 |
|  |  | Gopalganj | 91.08 | 5,994 | 0.00 | 0 | 4.88 | 321 | 4.04 | 266.00 | 6,581 |
|  |  | Katihar | 98.20 | 3,378 | 0.00 | 0 | 0.99 | 34 | 0.81 | 28.00 | 3,440 |
|  |  | Muzaffarpur | 96.74 | 9,994 | 0.00 | 0 | 0.87 | 90 | 2.39 | 247.00 | 10,331 |
|  |  | Purnia | 98.79 | 9,153 | 0.00 | 0 | 0.17 | 16 | 1.04 | 96.00 | 9,265 |
|  |  | Samastipur | 92.40 | 10,781 | 0.00 | 0 | 4.58 | 534 | 3.03 | 353.00 | 11,668 |
