# Supplementary material for: Impact of IRS: Four-years of entomological surveillance of the Indian Visceral Leishmaniases elimination programme
Source: PLoS Negl Trop Dis. 2021 Aug 9;15(8):e0009101. doi: 10.1371/journal.pntd.0009101 (PMC8376195; doi:10.1371/journal.pntd.0009101)
Supplement: S3 Table — (DOCX) [file pntd.0009101.s003.docx]

| **Year** | **IRS Round** | **District** | **Cattle sheds** | | | | | | | | |
| --- | --- | --- | --- | --- | --- | --- | --- | --- | --- | --- | --- |
|  |  |  | **Sprayed (Complete)** | | **Sprayed (Partial)** | | **Refused** | | | **Locked** | **Total** |
|  |  |  | **%** | **n** | **%** | **n** | **%** | **n** | **%** | **n** |  |
| **2017** | **1** | East Champaran | 98.44 | 2,706 | 0.00 | 0 | 1.09 | 30 | 0.47 | 13 | 2,749 |
|  |  | Godda | No Data | | No Data | | No Data | | No Data | | 0 |
|  |  | Gopalganj | 98.63 | 432 | 0.00 | 0 | 1.37 | 6 | 0.00 | 0 | 438 |
|  |  | Katihar | 99.83 | 1,199 | 0.00 | 0 | 0.00 | 0 | 0.17 | 2 | 1,201 |
|  |  | Muzaffarpur | 97.24 | 3,587 | 0.00 | 0 | 1.41 | 52 | 1.36 | 50 | 3,689 |
|  |  | Purnia | 98.39 | 2,378 | 0.00 | 0 | 0.46 | 11 | 1.16 | 28 | 2,417 |
|  |  | Samastipur | 97.44 | 2,322 | 0.00 | 0 | 1.64 | 39 | 0.92 | 22 | 2,383 |
|  | **2** | Darjeeling | 100.00 | 50 | 0.00 | 0 | 0.00 |  | 0.00 |  | 50 |
|  |  | East Champaran | 68.31 | 194 | 0.00 | 0 | 27.46 | 78 | 4.23 | 12 | 284 |
|  |  | Godda | No Data | | No Data | | No Data | | No Data | | 0 |
|  |  | Gopalganj | 99.29 | 1,403 | 0.00 | 0 | 0.71 | 10 | 0.00 | 0 | 1,413 |
|  |  | Muzaffarpur | 97.33 | 3,131 | 0.00 | 0 | 1.15 | 37 | 1.52 | 49 | 3,217 |
|  |  | Purnia | 97.76 | 3,406 | 0.00 | 0 | 1.18 | 41 | 1.06 | 37 | 3,484 |
|  |  | Samastipur | 96.95 | 2,096 | 0.00 | 0 | 1.62 | 35 | 1.43 | 31 | 2,162 |
| **2018** | **1** | Darjeeling | 100.00 | 200 | 0.00 | 0 | 0.00 | 0 | 0.00 | 0 | 200 |
|  |  | East Champaran | 98.95 | 3,006 | 0.00 | 0 | 0.89 | 27 | 0.16 | 5 | 3,038 |
|  |  | Godda | No Data | | No Data | | No Data | | No Data | | 0 |
|  |  | Gopalganj | 99.82 | 1,666 | 0.00 | 0 | 0.18 | 3 | 0.00 | 0 | 1,669 |
|  |  | Katihar | 100.00 | 715 | 0.00 | 0 | 0.00 | 0 | 0.00 | 0 | 715 |
|  |  | Muzaffarpur | 95.96 | 1,354 | 0.00 | 0 | 1.77 | 25 | 2.27 | 32 | 1,411 |
|  |  | Purnia | 97.59 | 2,920 | 0.00 | 0 | 1.37 | 41 | 1.04 | 31 | 2,992 |
|  |  | Samastipur | 95.99 | 1,770 | 0.00 | 0 | 2.77 | 51 | 1.25 | 23 | 1,844 |
| **2019** | **1** | Godda | No Data | | No Data | | No Data | | No Data | | 0 |
|  |  | Gopalganj | 95.53 | 982 | 0.00 | 0 | 1.95 | 20 | 2.53 | 26 | 1,028 |
|  |  | Purnia | 96.72 | 2,772 | 0.00 | 0 | 1.64 | 47 | 1.64 | 47 | 2,866 |
|  | **2** | East Champaran | 95.89 | 3,706 | 0.00 | 0 | 1.60 | 62 | 2.51 | 97 | 3,865 |
|  |  | Godda | No Data | | No Data | | No Data | | No Data | | 0 |
|  |  | Gopalganj | 99.12 | 674 | 0.00 | 0 | 0.44 | 3 | 0.44 | 3 | 680 |
|  |  | Katihar | 97.48 | 310 | 0.00 | 0 | 0.94 | 3 | 1.57 | 5 | 318 |
|  |  | Muzaffarpur | 97.88 | 2,171 | 0.00 | 0 | 0.77 | 17 | 1.35 | 30 | 2,218 |
|  |  | Purnia | 99.07 | 1,590 | 0.00 | 0 | 0.37 | 6 | 0.56 | 9 | 1,605 |
|  |  | Samastipur | 95.01 | 1,655 | 0.00 | 0 | 2.35 | 41 | 2.64 | 46 | 1,742 |
