# Supplementary material for: Impact of IRS: Four-years of entomological surveillance of the Indian Visceral Leishmaniases elimination programme
Source: PLoS Negl Trop Dis. 2021 Aug 9;15(8):e0009101. doi: 10.1371/journal.pntd.0009101 (PMC8376195; doi:10.1371/journal.pntd.0009101)
Supplement: Attachment — Submitted filename: 20210517_response to reviewers.docx [file pntd.0009101.s004.docx]

To whom it may concern:

We would like to thank the reviewers for their comments and have detailed our responses below.

Reviewer #1: - It should be “confidence level” when the authors stated the following at the beginning of page-4 of the manuscript: “Eight hundred households were targeted for each spray round in
- each of these districts assuming a 95% confidence interval, 5% absolute precision and 50% expected coverage based on the most conservative measure..” Did the authors also considered design effect, as they were dealing with clusters?

This has been changed from “confidence interval” to “confidence level”.

The sample size estimation exercise was carried out to attain a sample size that would provide robust district-level estimate. A conservative district-level sample size (i.e., the largest sample size irrespective of the extent of error), 0.5 was taken as an expected frequency of error for the calculations. The process considered cluster effects as well using the design effect of two. We have modified the methods to include the cluster effect.

Reviewer #2: It is a descriptive study and no hypothesis was aimed to be tested.
Accepted

Reviewer #3: Objectives articulated, Study design appropriate, population clearly described, sample size sufficient, correctly analysed and ethically cleared. Authors must clarify in which lab HPLC was performed. There is a defect that they had not send few samples to WHO recommended lab for cross check.

All the partners and institutes where the work was carried out are listed in the authorship. We followed the WHO “Monitoring and Evaluation Tool Kit for indoor residual spraying. Kala-azar elimination in Bangladesh, India and Nepal.” <https://www.who.int/tdr/publications/documents/irs_toolkit.pdf> This does not mention the need to cross reference samples with a WHO laboratory.

--------------------

 Results

-Does the analysis presented match the analysis plan?
-Are the results clearly and completely presented?
-Are the figures (Tables, Images) of sufficient quality for clarity?

Reviewer #1: Results are well analyzed and presented.
Accepted
Reviewer #2: Yes, study results matched with the analysis plan. Results are presented adequately. However, discussion section needs better interpretation of the study results.

The discussion has been strengthened. Please see new manuscript and notes below.

Reviewer #3: Analysis match and results clearly presented.
Number of Table numbered incorrectly, figures have some defects. Correction of these defects have been suggested.

Numbering of tables and figures has been corrected and suggested changes been made.

--------------------

Conclusions
-Are the conclusions supported by the data presented?
-Are the limitations of analysis clearly described?
-Do the authors discuss how these data can be helpful to advance our understanding of the topic under study?
-Is public health relevance addressed?

Reviewer #1: - The following statement at the bottom of page-9 is not correct.
“While a range of different vector control interventions for P. argentipes have been tested the only one to show success to date is IRS, limiting the options for vector control to a single intervention.” Other vector control measures, for example ITN, also work effectively in community settings, as shown in different studies.”

We have changed this to recognise the use of ITNs in certain communities with references and restructured the statement to one of IRS being the intervention of choice in India.

“*While a range of different vector control interventions including ITNs for P. argentipes have been evaluated and can control VL in some communities (34, 35), IRS has had the greater impact making it the preferred vector control tool in India (8, 9, 16, 36).”*

Reviewer #2: Study limitation is missing.

This has now been added at the end of the results section.

Study data found decline in LD infection rate among sand fly against historical data, but the study did not have its own baseline information regarding this. Therefore it is hard to accept that the decline of LD infection among SF was due to IRS. Other possible factors must be discussed.

We agree with this comment, the reduction in LD infection in SF will be a combination of vector control and improved case detection and management. Both of these will have impacted on the transmission. While we have alluded to this elsewhere in the manuscript, we have strengthened that here as well. Case management will have reduced the human reservoir of *P. argentipes.*

*“Across all three states between 2017 and 2019 we detected only 0.03% of P. argentipes infected with L. donovani in-IRS villages. This is significantly lower than previous studies where P. argentipes infected with L. donovani ranged from 0.85% to 32% (48-52). This low level of infection being detected is due to both the impact of the case detection and treatment reducing the human reservoir of L. donovani (53) and the impact of IRS that will reduce the abundance and age of the vector (54, 55). The low abundance and low level of infection of P. argentipes suggests that active transmission in the region is now low, which is evidenced in the reduction in cases (Table 4).”*

The study results failed to demonstrate that improved IRS activities in the study areas succeed in drooping SF densities as the differences of SF densities between IRS and non-IRS areas were insignificant.

As this is a surveillance programme and not designed as a cluster randomised trial, it is not valid to statistically compare numbers directly between IRS and non-IRS sites. We have also highlighted this issue of close proximity between IRS and non-IRS villages in the manuscript. There may well have been a spill over effect of IRS into the non-IRS sites that we cannot account for here.

The WHO “Monitoring and Evaluation Tool Kit for indoor residual spraying. Kala-azar elimination in Bangladesh, India and Nepal.” <https://www.who.int/tdr/publications/documents/irs_toolkit.pdf> referenced here uses the non-IRS sites to determine any seasonal or social effects that could impact on the IRS. We were not able to determine any from this data.

Therefore, does the national program need to continue IRS which is a big burden for the Government like India in relation its cost, logistics, HR and infrastructure?

We agree that there is a need now for the VL programme to reorientate itself as outlined in the WHO guidelines. In fact, this is what the programme is currently working towards in 2021/22 with increased investment in surveillance, however, these activities have been hindered by the COVID pandemic (not part of this manuscript).

We have added the need under WHO to orientate now away from complete IRS and improved surveillance as the end paragraph as a conclusion.

*“Due to the improved case detection, treatment and vector control, transmission of L. donovani by P. argentipes in India is currently low, which is evidenced in the reduction in cases. This success suggests that it is time for the programme to orientate in line with the WHO VL elimination guidelines (13, 15) to the consolidation phase. In this phase the total coverage by spraying is may no longer required. At the same time there needs enhanced surveillance is needed to detect increases in VL incidence or changes in the sand fly population so that ant potential disease resurgence will elicit a rapid response.“*

Reviewer #3: There is no separate column for conclusion. Limitations have not been described.
This publication will help scientists working in this field. This will also help policy makers of India, Nepal and Bangladesh to check their policy to eliminate Kala-azar.

Conclusion now added please see above comment.

--------------------

Editorial and Data Presentation Modifications?
Use this section for editorial suggestions as well as relatively minor modifications of existing data that would enhance clarity. If the only modifications needed are minor and/or editorial, you may wish to recommend “Minor Revision” or “Accept”. 

Reviewer #1: (No Response)
Accept
Reviewer #2: The need of IRS must be justified with adequate data, otherwise it has to be discouraged in the light of the study results.

We agree with this statement as the control programme has now reached its elimination phase there is a need for it to re-orientate how things will be done going forward. This has been added into the conclusion.

Reviewer #3: (No Response)
Accept
--------------------

<b>Summary and General Comments
Use this section to provide overall comments, discuss strengths/weaknesses of the study, novelty, significance, general execution and scholarship. You may also include additional comments for the author, including concerns about dual publication, research ethics, or publication ethics. If requesting major revision, please articulate the new experiments that are needed.

Reviewer #1: - One of the key elimination strategies was “integrated vector management and vector surveillance”. It did not focus on IRS only, as said in the Abstract and Introduction of the manuscript.

We agree that the guidelines do mention the concept of an IVM strategy, and this is in the discussion. However, the 2015 Operational Guidelines on Kala-azar Elimination by the Government of India focuses on IRS to achieve IVM <https://nvbdcp.gov.in/Doc/opertional-guideline-KA-2015.pdf> (section 4.4). In India, no other systematic vector control initiatives have been implemented, and the authors (which includes the MoH) reflects this in the manuscript.

- It would be better if the author says South Asia, instead of Indian subcontinent. It is no longer like that after the British left the region in 1947. They cleverly divided the region before leaving their beloved colony.

We have chosen to use “South Asia”, however, to avoid confusion on the geographic region we have kept the older term “Indian subcontinent” in brackets as many of the references including those from WHO and South Asian authors utilise this term. Appropriate changes have been made in text.

- The elimination target is not described correctly. Please have a look at the referenced resources. Although, these are clearly presented under Discussion.

The elimination target has been redefined for India as VL incidence of less than 1/1000 people at the sub-district (block).

- The elimination strategies mentioned are also incomplete and wrongly put vector control first.

The focus of this paper is on vector control and makes that its central theme, however, the manuscript acknowledges that it is the combined efforts of IRS and improved case management that is allowing the programme to move towards elimination. While it is not possible to say which has contributed more to the success of the elimination programme, we have strengthened the acknowledgement of case management.

- The authors should have made it clear that DDT is (was) used in India only, and has been banned from using for vector control in other South Asian countries like Bangladesh. The authors cited reference (16) in the Introduction and mentioned about the usefulness of DDT. But in that study, only India used DDT. Neither Bangladesh nor Nepal study sites used DDT as intervention. The authors should have read the actual article before citing it in their manuscript. Or they might have overlooked that willingly, which is unscientific.

This was a typo and has been altered to “insecticide-based IRS”.

Reviewer #2: This is an observational study on IRS in relation to the National VL elimination program in India. The study had 8 sites for data collection. In India more than 600 blocks are endemic for VL. So inclusion of 8 sites represents only 1.6% of the all VL endemic areas which is insufficient regarding sample size calculation.

As Reviewer 2 kindly points out this is an observational study based on the 8 VL sentinel surveillance sites and as noted by other reviewers this is a descriptive paper. The manuscript is not based on data from a cluster randomised trial that would have required a much higher number of sites at a prohibitive cost.

Throughout the manuscript we have based findings at sentinel sites level only with implied interpretation for the wider elimination programme. These assumptions can be made as the number of blocks that had remaining to reach elimination in 2020 was just 17, with all blocks having received improved case management and vector control.

Over sprayed filter papers with IRS for monitoring demonstrated that those were unblinded for spray men making them unacceptable for validation of spray quality.

As only a subset of the papers was tested the effect is that all the other papers are blind, spray operators not knowing which papers would be tested.

IRS coverage among IRS areas was consistently low than that recommended by the WHO. Further lack of differences in SF densities between IRS and non-IRS areas makes this intervention questionable for its further continuation.

We agree with this statement as the control programme has now reached its elimination phase there is a need for it to re-orientate how things will be done going forward. This has been added into the conclusion.

Authors shall highlights these findings in the discussion and make adequate recommendation.

Reviewer #3: These are the general suggestion/modifications/ questions to be addressed by authors to improve the paper.
Authors Summary:
Last line: VL elimination target of 1 case per 10,000 at the sub-district level.
Suggestion: India has no structure like sub-district; sub district level is target for Bangladesh. It should be less than 1 case per 10,000 population at…….. level.

This has been altered to show “block” which is the sub-district level used in India for the target of VL elimination.

Method
Each site had at least 1 new VL case per 10,000 capita per year at sub-district (block) level.
Q a: What do you mean by 10,000 capita per year?

We have changed “capita” to “persons” to keep this statement in line with the rest of the manuscript. Essentially, this statement defines sites being selected that had not already reached the elimination target as IRS would be halted and the surveillance programme aimed at monitoring IRS.

Q b: What do you mean by sub-district level? Sub-districts are in Bangladesh. Please check tripartite agreement done in 2005 between Bangladesh, India, and Nepal.

Sub-district implies the level below district. We have indicated this to mean the “block” level in India as this is used to measure the elimination target against. We have gone on to use block instead of sub-district in the text to avoid confusion. This is in line with the Regional Strategic Framework for Elimination of Kala-azar from the South-East Asia Region (2005-2015) WHO-SEA 2005.

Quality assurance:
Your line: and stored at -4 0C until analysis following IRS activities.
Suggestion: You must have noticed that impregnated papers were to be stored at room temperature, similarly to keep at -4 oc is not recommended. Hence remove these words.

As the methods section is a description of what we did allowing another person to repeat the experiment as described, as opposed to what might have done. We have not made the change.

Filter papers are indeed dried at room temperature and can even be stored at room temperature for short periods. This is useful as many field settings do not have the ability to store papers at lower temperatures. However, room temperature can be highly variable depending on the location and time of year.  Therefore, when feasible storing filters at 4^0^C is preferred since it provides a stable temperature and additional chemical stability for longer periods.

It should be noted that WHO does not recommend against storing these samples at 4^0^C only that “For both ITNs and IRS, samples are placed individually in labelled, aluminium foil before dispatch to a pesticide analytical laboratory.” Ref: <https://apps.who.int/iris/bitstream/handle/10665/69296/WHO_CDS_NTD_WHOPES_GCDPP_2006.3_eng.pdf?sequence=1>

You: HPLC was performed…..
Question: In which lab it was performed? Name the lab.

Whether some sample was sent to another lab to cross check the results?

You must have read that different labs had different results. Hence, few samples must be cross checked from WHO recommended lab.

All the partners and institutes where the work was carried out are listed in the authorship. We followed the WHO “Monitoring and Evaluation Tool Kit for indoor residual spraying. Kala-azar elimination in Bangladesh, India and Nepal.” <https://www.who.int/tdr/publications/documents/irs_toolkit.pdf> This does not mention the need to cross reference samples with a WHO laboratory.

Insecticide Susceptibility Assays
You: and Abbott’s formula applied to correct for control mortality
Suggestion: a. For readers provide either Abbott’s formula or the reference.
b. Abbott’s formula is not used to correct control mortality but it is used to correct test mortality if control mortality is within 20%. Correct the text accordingly.
Phlebotomus argentipes abundance.

This sentence was changed to “*Controls were performed for each test using appropriate papers, and Abbott’s formula was applied where necessary (28)*.” And the reference for Abbots formula added.

You: by morphological criteria from established taxonomic keys (28). Suggestion: Correct the reference in reference section.

Reference is now corrected to.

*Kalra NL, Bang YM. Manuals on Entomology in Visceral Leishmaniasis. New Delhi: WHO, SEARO; 1988.*

Results 
Indoor residual spraying data
You:. …spray operators reporting over 80% coverage of households and cattle sheds covered with alpha-cypermethrin at 0.25g/m2 IRS. 
Suggestion: correct the dose according to methodology section.

Corrected so that all measurements are stated the same

IRS coverage as measured from the survey data:
You: In Bihar, consistently low-level of complete coverage over the four years was observed in Samastipur (49-61%).
Suggestion: What were the causes? Discusses them in discussion section. Provide your suggestions to overcome the problem.

This was due to homeowner’s preference to which areas of the home may be sprayed. This has been added into the discussion along with the improved engagement with homeowners to have all of the structure sprayed.

Quality assurance:
A total of 642 houses that received IRS across the eight sites were included in the QA surveys. Four filter papers per wall were affixed in the bedroom prior to IRS and recovered afterwards. A total of 3,050 Whatman filter papers were analysed by HPLC over three years (2017-2019). In 2017, all 2,140 filter papers collected from field surveys were analysed. In subsequent years a random sample of 20% of houses from each sentinel site were analysed.

Question: You had taken 642 houses and 4 filter papers per room (642 x 4= 2568). Then from where you have collected 3050 samples for HPLC)? If your second line is considered 20% of 642 houses is 128 x 4 walls = 512. Hence, total sample will be 2568+512= 3080. Justify and rewrite this paragraph.
You: Of the 2,944 filter papers tested from 2017 t002019….

There were some operational losses of papers which has been explained in the manuscript.

Question: Now from where this number arrived? Which of the samples were discarded?

There were some operational losses of papers which has been explained in the manuscript.

You: In East Champaran low quality spraying increased annually from 48.3% in round 1 of 2017 to 70% in round 2 of 2019. Suggestion: Re-frame the sentence to clear the meaning (low quality increased or quality improved?).

This has been made clearer. “*In East Champaran the quality of spraying declined annually from 48.3% under spray in round 1 of 2017 to 70% under spray in round 2 of 2019*.”

You: No resistance was detected to the other insecticides tested, Table 2 (21).
Suggestion: a. Where is your Table 1 (in the text Table 1 is missing).
b. In results section your findings should be explained, You should not place any reference like 21.

The reference has been removed as the statement “*No resistance was detected to the other insecticides tested, Table 2”,* only applies to the data in this manuscript.

Noted that table and figure numbers were incorrect in places this has been corrected throughout the text.

You: in IRS villages, and here we report a reduction to 0.75 P. argentipes/trap/night for the same time period in 2019. (21), in IRS villages, and here we report a reduction to 0.75 P. argentipes/trap/night for the same time period in 2019.
Suggestion: Duplication of line.

Duplication deleted.

Your Table 2: During 2016 and 2019, why mortality in low concentration is high for Alpha cypermethrin? Discuss.

Considering that we are using wild caught sand flies, as outlined in the manuscript, that are not standardised for age, pre-exposure to insecticides or other stressors, these minor fluctuations in mortality are expected and within the experimental tolerance.

Figure 2. Percentage of household structures that were completely sprayed in each IRS round as reported by; A spray operators in all 8 sites, and B sentinel sites surveys. R1= Round one R2=Round two. Solid colors represent complete spray and lined colors partial spray. 
Question a: you have 8 sentinel sites. 1. One bar is missing in all the cases except 2018 R1.
b. In bar there are colors but you have not correlated the colors to different sites.

The manuscript states that the household survey was carried out in Bihar and Jharkhand only, hence there are only 7 sites reported in the graph. We have made this clearer in the methods section by including the statement *(West Bengal was not included in this survey).*

While the colours were matched some are very similar. This has been improved in the new graphs for clarity.

2018 R1 only has 7 bars.

Fig 3: has been labelled as Fig 2.

Figure numbers have been corrected throughout.

Suggestion: This fig. is related to dose. Colors are indicated as spray/target /spray. Correct them.

The graph shows percentage of household structures that are sprayed on target, under sprayed or over sprayed. While this is related to dose of insecticide the measure is percentage. This has been qualified in the Fig title giving the reader definitions of *“% over sprayed- >30 mg/m^2^; % on target-20-30mg/m^2^; % under sprayed - <20 mg/m^2^ alpha-cypermethrin*.”

Figure 4: Aggregated P. argentipes abundance 
Question: Why the abundance of P. argentipes is high in IRS in comparison to non-IRS in July 2017? Discuss.

The actual numbers of sand flies currently being caught are extremely low across all sites. While we have used non-IRS villages, they border IRS village and the impact on the sand fly populations across village borders cannot be accounted for. This has been discussed more in the manuscript.

--------------------

PLOS authors have the option to publish the peer review history of their article (<a href="[https://journals.plos.org/plosntds/s/editorial-and-peer-review-process#loc-peer-review-history](https://protect-eu.mimecast.com/s/5inECj0jKSnQRzmhxx4Kj?domain=journals.plos.org)" target="_blank">what does this mean?</a>). If published, this will include your full peer review and any attached files.<br><br>

If you choose “no”, your identity will remain anonymous but your review may still be made public.<br><br>

<b>Do you want your identity to be public for this peer review?</b> For information about this choice, including consent withdrawal, please see our <a href="[https://www.plos.org/privacy-policy](https://protect-eu.mimecast.com/s/0guDCkrkLiOz5DBF0Izzz?domain=plos.org)" target="_blank">Privacy Policy</a>.

Reviewer #1: No

Reviewer #2: No

Reviewer #3: Yes: Prof. Dr Murari Lal Das


Figure Files:
While revising your submission, please upload your figure files to the Preflight Analysis and Conversion Engine (PACE) digital diagnostic tool, [https://pacev2.apexcovantage.com](https://protect-eu.mimecast.com/s/eahXCl2lgU2NXWEcNJDO1?domain=pacev2.apexcovantage.com). PACE helps ensure that figures meet PLOS requirements. To use PACE, you must first register as a user. Then, login and navigate to the UPLOAD tab, where you will find detailed instructions on how to use the tool. If you encounter any issues or have any questions when using PACE, please email us at [figures@plos.org](mailto:figures@plos.org).
 
Data Requirements:
Please note that, as a condition of publication, PLOS' data policy requires that you make available all data used to draw the conclusions outlined in your manuscript. Data must be deposited in an appropriate repository, included within the body of the manuscript, or uploaded as supporting information. This includes all numerical values that were used to generate graphs, histograms etc.. For an example see here: [http://www.plosbiology.org/article/info:doi/10.1371/journal.pbio.1001908#s5](https://protect-eu.mimecast.com/s/RhoYCmYmjSjNWVlTwwlsM?domain=plosbiology.org).
 
Reproducibility:
To enhance the reproducibility of your results, we recommend that you deposit your laboratory protocols in [protocols.io](https://protect-eu.mimecast.com/s/UyjNCng0kU7Nm4DIzGCWK?domain=protocols.io), where a protocol can be assigned its own identifier (DOI) such that it can be cited independently in the future. Additionally, PLOS ONE offers an option to publish peer-reviewed clinical study protocols. Read more information on sharing protocols at [https://plos.org/protocols?utm_medium=editorial-email&utm_source=authorletters&utm_campaign=protocols](https://protect-eu.mimecast.com/s/AjWKCojolfr3vAYsmHvZ6?domain=plos.org)

__________________________________________________
In compliance with data protection regulations, you may request that we remove your personal registration details at any time. (Use the following URL: [https://www.editorialmanager.com/pntd/login.asp?a=r](https://protect-eu.mimecast.com/s/1LQUCpkpmIn3Agoh99x06?domain=editorialmanager.com)). Please contact the publication office if you have any questions.
