# Supplementary material for: Impact of IRS: Four-years of entomological surveillance of the Indian Visceral Leishmaniases elimination programme
Source: PLoS Negl Trop Dis. 2021 Aug 9;15(8):e0009101. doi: 10.1371/journal.pntd.0009101 (PMC8376195; doi:10.1371/journal.pntd.0009101)
Supplement: Attachment — Submitted filename: 2021_Impact of IRS_VL_elimination.docx [file pntd.0009101.s006.docx]

To Whom It May Concern:

We are grateful to the reviewer’s comments and have made the following changes based on their suggestions.

We have edited the manuscript as suggested by the reviewer.

I Chaudhuri’s affiliation has been added

Reference 46 was corrected.

Thank you

Mike Coleman
